# The shape of density dependence in fragmented landscapes explains an inverse buffer effect in a migratory songbird

**DOI:** 10.1038/s41598-017-15180-4

**Published:** 2017-11-06

**Authors:** Caz M. Taylor

**Affiliations:** Department of Ecology and Evolutionary Biology, Tulane University, New Orleans, LA 70118 USA

## Abstract

It is well known that forest fragmentation reduces fecundity in several avian species, including wood thrush, *Hylocichla mustelina*, a migratory songbird that has been declining for several decades. However, I found that landscape-scale density in wood thrush was lower and population declines steeper in higher quality, less-fragmented landscapes (an *inverse buffer effect*) than in poor quality landscapes. These patterns suggest that wood thrush was not limited by availability of breeding habitat but that declines were primarily driven by non-breeding season events. A two-season model predicts that if this hypothesis is correct, breeding population trends will be negatively related to the strength of density dependence (*b’*) in the breeding season. To test this, a site-dependence model was used to construct fecundity-density curves and showed that landscape fragmentation affected the shape of density dependence. In good quality landscapes, the onset of strong density dependence was much more abrupt than in poorer quality landscapes and the realized strength of density dependence, *b’*, was lower in good quality landscapes. Population trends were negatively associated with *b’*, providing support for the non-breeding limitation hypothesis. The combination of the negative associations of trends with *b’* and *b’* with landscape quality explain the inverse buffer effect.

## Introduction

Forest fragmentation has been shown to be a major component of breeding habitat quality for several forest-dwelling bird species that experience lower fecundity in smaller forest patches or more fragmented landscapes^1^. Forest fragmentation in North America has been put forward as a possible cause of decline for neotropical migratory species^1^. The mechanisms that reduce fecundity are that nests in small forest patches or close to forest edges are more vulnerable to predators and to parasitism from nest parasites, such as the brown-headed cowbird, *Molothrus ater* ^1^. Landscape effects on the abundance of nest parasites and predators interact with forest fragmentation to influence fecundity at larger scales^2^. Fecundity in birds is also affected by density and it has been shown that density-dependent processes can operate at multiple scales^3^. To understand the landscape-scale effects of fragmentation, we need to assess how fragmentation interacts with density dependence.

One particularly well-studied species with respect to forest fragmentation is the wood thrush, *Hylocichla mustelina*, a neotropical migrant that winters in central America and breeds throughout out the eastern USA. Multiple studies have shown substantially lower fecundity (nest survival or number of fledged young per female) in more fragmented landscapes or in smaller forest patches^2,4–7^. Analysis of the North American Breeding Bird Survey (BBS) shows that breeding populations of this species have declined in recent decades at an overall rate of ~ −2% year^−1^ but with considerable variation in trends across the breeding range^8^. Primary causes of these declines, especially at the species-level, are still being debated. The strong sensitivity of fecundity in wood thrush to forest fragmentation as well as correlations between local trends and breeding habitat loss as well as the strong contribution of fecundity to spatial or temporal variation in growth rates suggest that declines are driven in large part by breeding habitat loss or fragmentation and that wood thrush population growth is limited by availability of breeding habitat or productivity^9–11^. However a full annual cycle, spatial network population that allows for density dependence in both non-breeding survival and fecundity indicates that, while breeding habitat loss and fragmentation have affected regional population changes, the primary driver of the species-wide population declines in wood thrush is non-breeding habitat loss and/or fragmentation^12^.

A two-season model, developed by Sutherland^13,14^, leads to predictions that can allow determination of the driver of large-scale population changes. The model assumes that the population size of a migratory animal is regulated by density-dependent processes in both breeding season (where fecundity-density curves are decreasing) and non-breeding season (where mortality-density curves are increasing). Events such as habitat loss, gain, degradation, improvement, or climate changes cause shifts in these seasonal curves resulting in a change to the size of the population. If a population is primarily limited by factors such as the availability or condition of habitat in one season, then an event (e.g., loss of habitat) in the limiting season will cause a large shift relative to an event of the same magnitude in the non-limiting season. Sutherland’s model showed that the expected magnitude of decline (or increase) in population size, $|{\rm{\Delta }}n|$, will be related to the ratio of the strength of density-dependent regulation in breeding (*b*′) to the strength of density dependence in non-breeding seasons (*d*′). Specifically, $|{\rm{\Delta }}n|$ will be related to the magnitude of the fecundity-density shift, $|{\rm{\Delta }}F|$, multiplied by *b*′/(*b*′ + *d*′) added to the magnitude of the mortality-density shift, $|{\rm{\Delta }}M|$, multiplied by *d*′/(*b*′ + *d*′) (Fig. S1)^13,14^. This leads to predictions that can be tested by examining population trends and strength of density dependence in only one season (in this study, the breeding season). Specifically, if population changes are driven primarily by breeding season events ($|{\rm{\Delta }}F|$ is large compared to $|{\rm{\Delta }}M|$), the model predicts that population trends in breeding regions across the breeding range will be positively related to *b*′, whereas if population changes are driven primarily by non-breeding season events ($|{\rm{\Delta }}F|$ is small compared to $|{\rm{\Delta }}M|$), the model predicts that $|{\rm{\Delta }}n|$ in breeding regions will be negatively related to *b*′. No relationship between $|{\rm{\Delta }}n|$ and *b*′ would be expected in a population that is limited by habitat availability in both seasons. To test these predictions for wood thrush, I estimated trends and density from the North American Breeding Bird Survey (BBS)^8,15^ in breeding landscapes with varying landscape quality (i.e., levels of fragmentation). I then modeled the shape of the fecundity-density curves in each landscape and calculated the strength of density dependence (*b*′) as the magnitude of the slope of the fecundity-density curve at the estimated population density.

Although there are several previous studies that estimate the shape of density-dependent relationships^16–20^, there are very few that demonstrate how extrinsic factors mediate the strength or alter the shape of density dependence^21,22^. Many previous studies have estimated the shape of density dependence in overall *per-capita* growth rate (*pgr*) by analyzing time-series of population size. These studies often find evidence of density-dependence by showing that the *pgr* of populations declines with increasing density. However the shapes of density-dependent growth rates are highly variable, even among taxa with similar life histories, ranging from convex (shallow declines in growth rates at low densities but steep declines at high densities) to linear to concave (steep declines in growth rates at low densities but shallow declines at high densities)^17–19^. An approach to estimate the shape of density dependence for a particular component of *pgr*, such as fecundity or survival, has been to conduct or review field studies that measure the fecundity or survival in populations of different densities. These studies also often show that such vital rates decline with increasing density but small sample sizes and the degree of stochasticity often preclude an estimation of the exact shape of the relationship^22,23^. Another approach to estimate the shape of density-dependence on one vital rate, and the one used here, is to generate the shape of density dependence in a vital rate from a model of the mechanism of density dependence^24,25^.

For wood thrushes, which are known to be territorial on their breeding grounds, the expected mechanism of density dependence is site dependence^26^. Site dependence assumes that nesting sites are preferentially selected in order of quality (better quality first) so that as density increases, individuals occupy increasingly poorer quality sites causing a drop in overall fecundity. I built a site-dependent habitat selection model that assumes that, for a fixed population size (number of individuals), forest patches are preferentially occupied in order of decreasing size until each patch reaches a saturation density^24^. Fecundity-density curves can be generated from the model by repeatedly applying the model to a distribution of patches for a range of population sizes and calculating the average fecundity and density (individuals per unit area) for each population size. Thus, application of this model to a real landscape and species requires knowledge of the distribution of forest patch sizes within the landscape, an estimation of fecundity as a function of patch size, and an estimation of the saturation density, also potentially a function of patch size.

**Figure 1:**
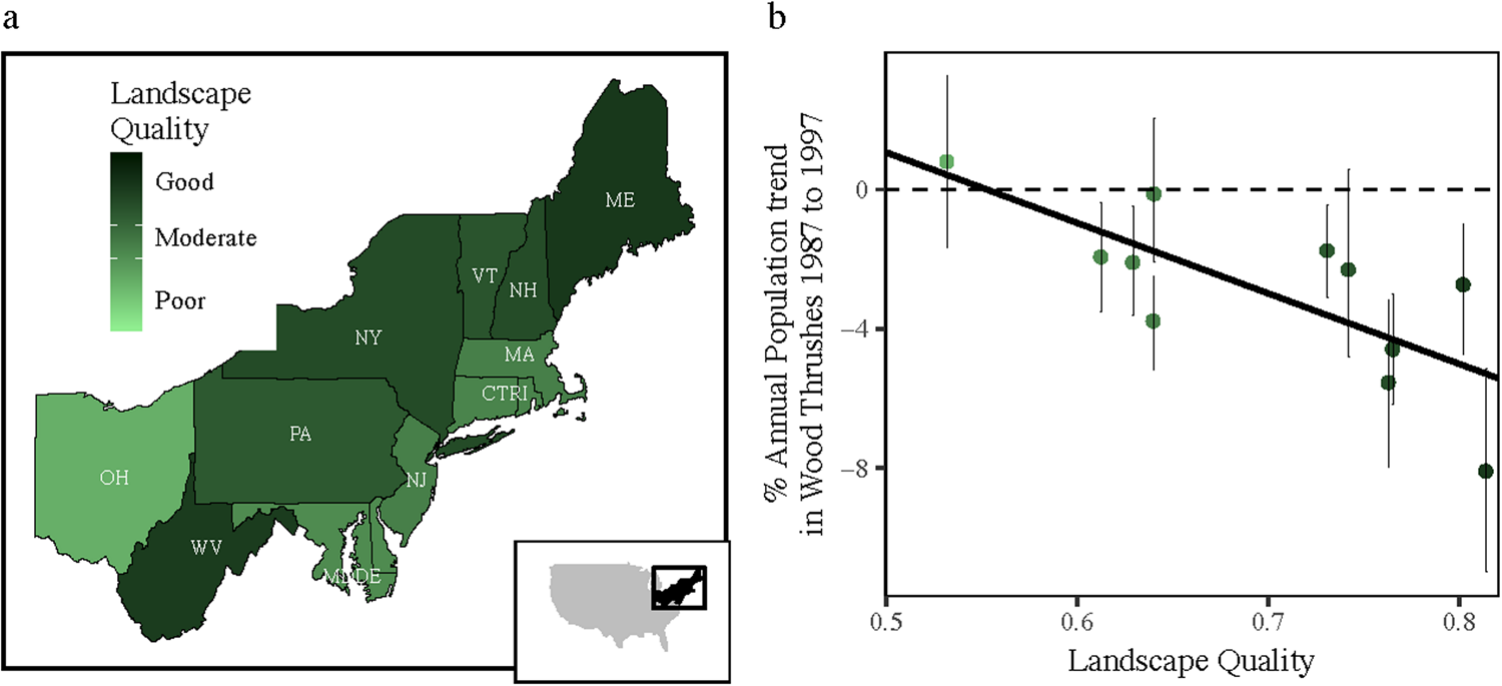
(**a**) Map of landscapes. Color scale represents landscape quality in early 1990s, the proportion of forest classified as core forest. Good quality is greater than 0.7 (PA VT NH NY WV ME), moderate quality is between 0.6 and 0.7 (MDDE, CTRI,MA,NJ), and poor quality is less than 0.6 (OH). (**b**) Population trends versus landscape quality shows an inverse buffer effect; large declines in good quality habitat. Error bars on trends are 2.5% to 97.5% confidence interval. All maps were generated using the maps package (version 3.1.1. https://CRAN.R-project.org/package=maps) in the R programming language^27^.

The site-dependence model was used to calculate fecundity-density curves for wood thrush in eleven landscapes in the Northeastern USA (Fig. 1a). Forest patch size distributions as well as *core* patch size distributions (core forest was defined as forest > 30 m from an edge) were available at the county level for thirteen states from a forest inventory study based on the 1992 National Land Cover Database (Fig. S2). State boundaries were used to define landscapes with each landscape being a single state except two where very small states were combined into neighboring states (Fig. 1a). For each landscape, *landscape quality* (*Q*) was quantified as the ratio of core to total forest area. A measure of total fecundity as a function of patch size was not available but expected average nest survival as well as saturation density as functions of patch size were modeled using data from a field study that was conducted over two years (1990–1991) in Pennsylvania (USA) within the Northeastern region^7^. The study showed an increasing relationship between patch area and nest survival, which I modeled with a saturating curve (Fig. S3a). Density was, surprisingly, higher in smaller patches than in larger patches and so saturation density was modeled as an inverse sigmoid curve (Fig. S3b). This pattern of higher density in smaller patches or more fragmented landscapes has been reported by many wood thrush studies^5–7,28,29^ and possible explanations are addressed in the discussion. Fecundity-density points were generated by applying the site-dependence model to the forest patch size distribution and a generalized Beverton-Holt (B-H) function was fitted^30^. One of the three parameters of the B-H function is *abruptness*, γ. If γ < = 1, the function is concave and if γ > 1, it is a reverse sigmoid shape, convex at densities up to an inflection point and concave above. A tipping point, defined as the density at which the strength of density-dependence (slope of curve) changed most rapidly in the convex part of the curve, was calculated for each landscape.

This goal of this study was to explore the role of fragmentation at the landscape level in demographic processes. In particular, I examined how fragmentation (landscape quality) was related to the shape and strength of density-dependence, the density of breeding birds, and population trends. The sign of the relationship between the strength of density dependence and the magnitude of population trends across landscapes was examined to test, based on Sutherland’s model^13,14^, whether the population trends, predominantly declines, were driven by processes, such as habitat loss, in the non-breeding versus breeding season.

## Results

Of the eleven landscapes, six were quantified as good quality (*Q* > 0.7), four as moderate quality (0.6 ≤ *Q* ≤ 0.7), and one as poor quality (*Q* < 0.6; Fig. 1a). Mean population trends in the decade centered around 1992 were negative in all but one of the eleven landscapes. In the poor quality landscape (OH), the mean population trend was slightly positive ( + 0.8% year^−1^) although not significantly different from zero. Mean trends in the moderate quality landscapes ranged from −3.8% to −0.13%. Mean trends in the good quality landscapes ranged from −8.1% in the highest quality landscape, ME, to −1.8%. More severe declines tended to occur in higher quality landscapes; there was a significant negative linear correlation between trend and landscape quality (*r*_9_ = −0.74; *p* < 0.01) and a significant positive linear correlation between absolute magnitude of trend and landscape quality (*r*_9_ = 0.67; *p* < 0.025) (Fig. 1b). I term this pattern an *inverse buffer effect* since, when the opposite pattern is observed (bigger declines or increases in lower quality habitat), it is termed a buffer effect and provides a possible regulating mechanism for a population^31,32^.

**Figure 2:**
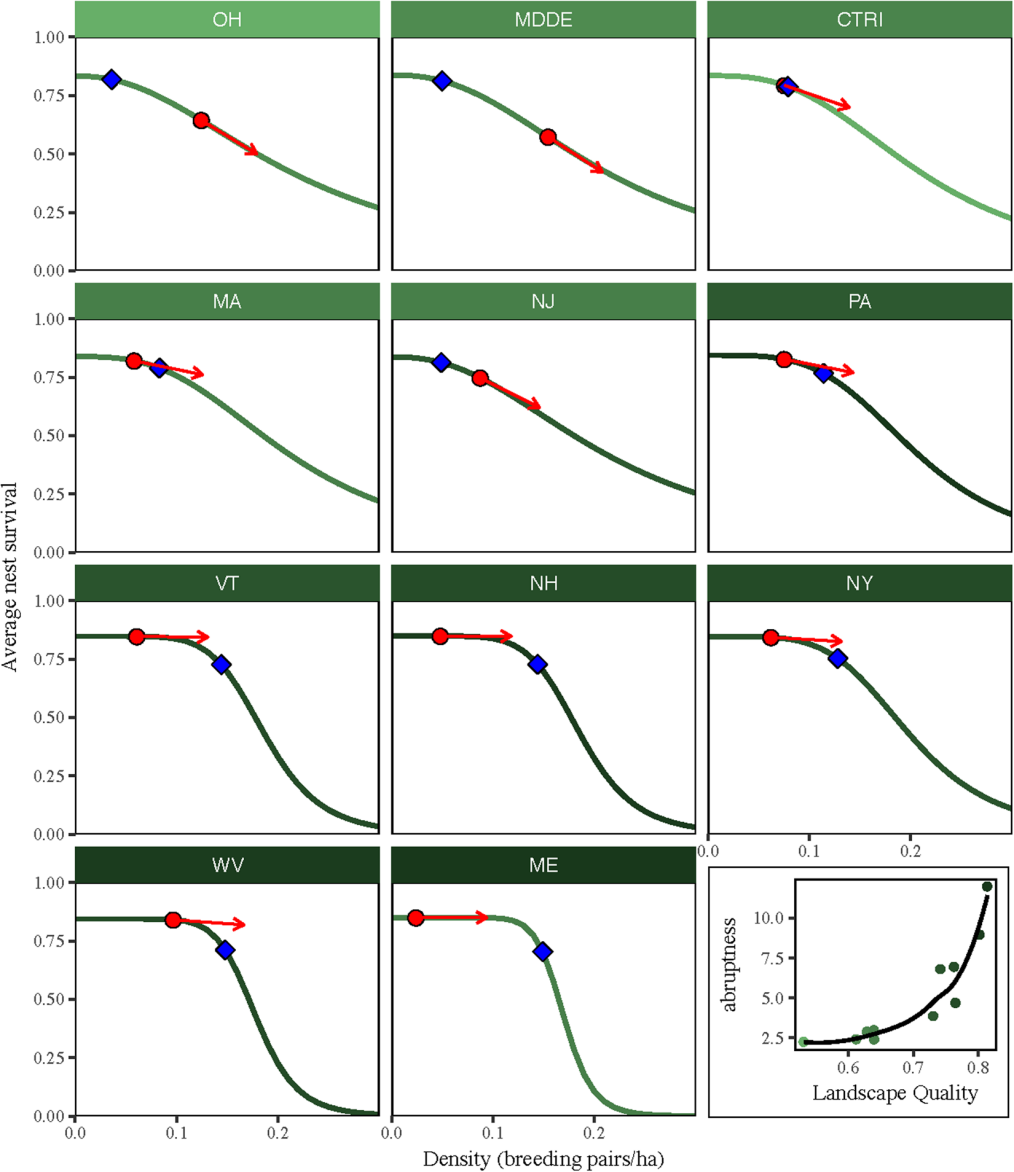
The shape of density-dependent nest-survival of wood thrush estimated as a generalized Beverton-Holt function^30^ from a site-dependence model for each landscape. Landscapes are arranged (left to right, top to bottom) from low to high quality. Red circles are estimated wood thrush density (breeding pairs ha^−1^) in 1992 and red arrows show the slope (strength of density dependence) at the estimated density. Tipping points (blue diamonds) are where the strength of density dependence is changing most rapidly. The bottom right panel shows how abruptness of the curves increases with increasing landscape quality with fitted loess curve.

I found that the shape of fecundity-density curves changed with forest fragmentation (Fig. 2). For all landscapes, fecundity was a convex function (abruptness γ > 1) of density at densities below 0.15–0.18 breeding pairs ha^−1^ and the abruptness of the curves increased with forest fragmentation (Fig. 2 lower right panel). In good-quality (less fragmented) landscapes, density dependence was weak at low densities but there was a dramatic strengthening of density dependence at high densities. In poor-quality (more fragmented) landscapes, fecundity was almost linearly related to density (Fig. 2). However, the tipping point, which ranged from 0.05 to 0.15 pairs ha^−1^, was also positively related to landscape quality such that the onset of stronger density dependence occurred at lower densities in poorer-quality landscapes.

**Figure 3:**
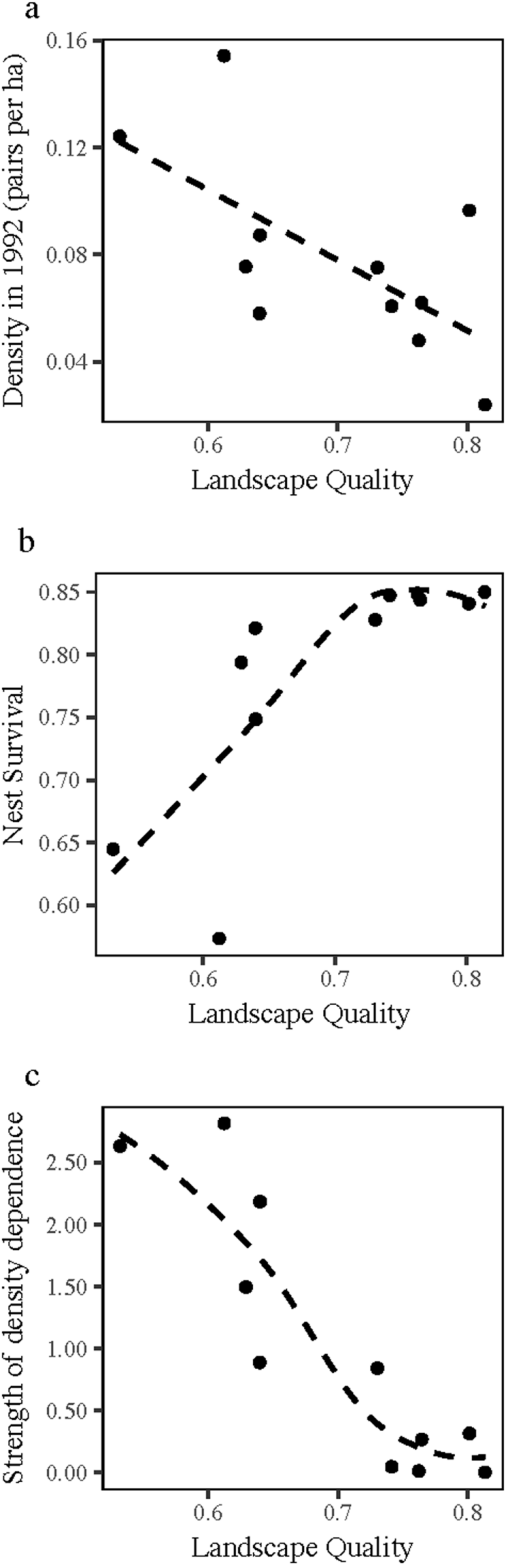
Relationships of landscape quality to demographic processes. (**a**) Density (pairs.ha^−1^) estimated from BBS decreases with landscape quality. Dashed line is linear correlation (*p*_9_ = −0.65; *p* < 0.03). (**b**) Average estimated nest survival increases with landscape quality. Dashed line is a fitted loess curve. (**c**) Estimated strength of density dependence is negatively related to landscape quality. Dashed line is a fitted loess curve.

Estimates of densities in each landscape from the breeding bird survey from 1992 were 0.02–0.15 breeding pairs ha^−1^ with density tending to be lower in good quality landscapes (Fig. 3a). Placing these densities on the fecundity curves showed that in good-quality landscapes, fecundity was below the tipping points, on the flatter parts of the curves (Fig. 2). The realized nest survival calculated from these points was positively related to landscape quality as expected (Fig. 3b) but the strength of density dependence, *b*′ (the absolute value of the slope of the curve at the estimated density), was negatively related to landscape quality (Fig. 3c).

**Figure 4:**
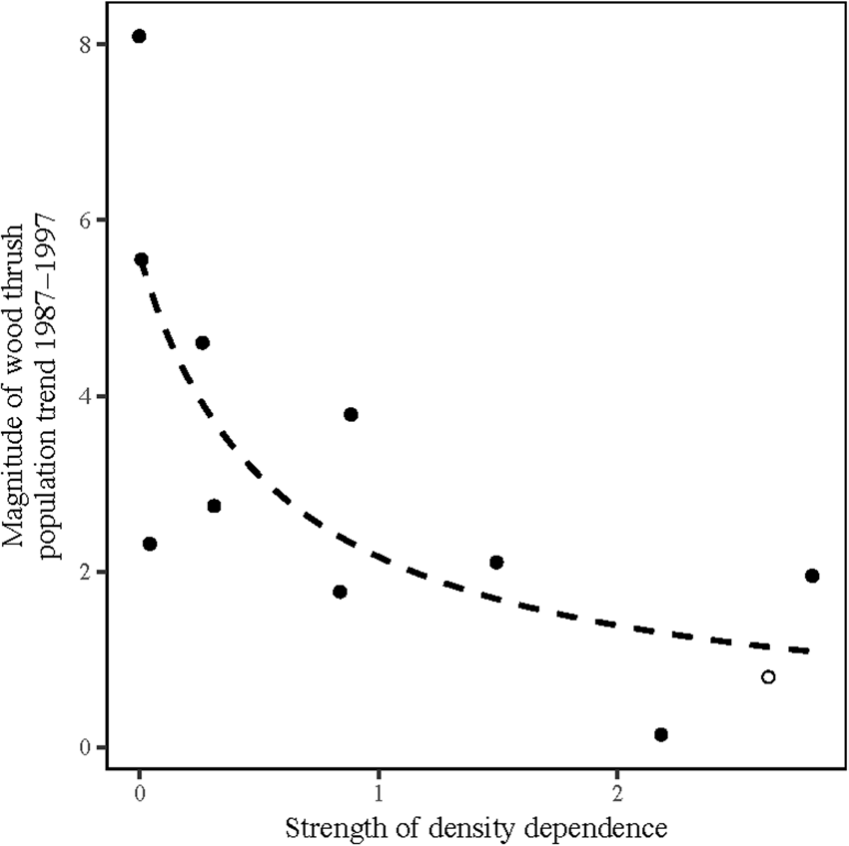
Magnitude (absolute value) of population trends, $|{\rm{\Delta }}n$|, in wood thrushes in 1992 decreases with strength of density dependence, *b*′. Dashed line is the best fit of the Sutherland model^13^ when declines are caused by non-breeding habitat loss, $|{\rm{\Delta }}n|=constant\ast {d}^{^{\prime} }/({b}^{^{\prime} }+{d}^{^{\prime} })$ assuming a fixed value for *d*′, which is the non-breeding strength of density dependence. Solid circles are negative trends (population declines) and the open circle is the positive trend in landscape OH.

There was a negative relationship between the magnitude of trends and the strength of density density-dependence, *b*′; the most severe population declines occurred where the population was most weakly regulated (Fig. 4). Based on Sutherland’s model (Fig. S1)^13,14^, this suggests that the overall declines in wood thrush at this time were primarily caused by habitat loss or other events in the non-breeding season.

The negative relationship of trend magnitude with b′ combined with negative relationship between b′ and quality combined produce the positive relationship of the magnitude of trends with landscape quality; the inverse buffer effect (Fig. 1b).

## Discussion

This study is among very few to demonstrate that the shape of density dependence can be affected by habitat quality. Here, I used a mechanistic model of habitat selection^24,26^ combined with data from a published field study^7^ to show that nest survival-density curves were abruptly convex in good quality landscapes but became less abrupt, almost linear, in poor quality landscapes. One other notable study^22^ showed a similar result experimentally. When densities of cactus bugs on host plants of varying qualities were manipulated, the probability of a juvenile surviving and not dispersing declined as a nonlinear decreasing function of density and the shape of this relationship shifted from concave to convex with increasing host-plant quality^22^. The shape of density-dependence may have implications for stability and persistence of populations. Mathematically, it has been shown that high abruptness in overall population growth rates tends to promote oscillations in long-term population dynamics^30^. For wood thrush, this may mean that populations in high quality landscapes are more likely to oscillate or be chaotic and thus be more vulnerable to stochastic events. However, the curves estimated here are only for density-dependent nest survival, one component of overall population growth rate, and population dynamics may be stabilized or counteracted by other processes.

I found that density at the landscape scale (birds per unit area of total forest in the landscape, estimated from the BBS) was higher in poorer quality landscapes (Fig. 3a). This pattern is consistent with results from many, smaller-scale field studies that show higher densities of wood thrush in smaller patches or in fragmented forest^5–7,28,29^. It has been suggested that higher density indicates a preference for smaller patches even though they are poorer quality (lower fecundity) and that forest fragmentation thus acts as an ecological trap for wood thrush and other forest bird species^5,33^. If true, this might provide an alternative explanation for the inverse buffer effect. However, preference for smaller patches would lead to positive density dependence – fecundity would increase with increasing density. Such a pattern has never been documented in wood thrush or other bird species and seems unlikely. It is well known that density is not always a good indicator of either quality or preference and there are several reasons why we might expect to see higher density in lower quality habitat^34^. For example, compared to smaller patches, larger patches may have higher species richness^35,36^, which potentially decreases the density of any one species due to interspecific competition^37^. Alternatively, for a highly territorial species, such as wood thrush, we might expect that good quality landscapes would be primarily occupied by dominant individuals. Such individuals may be able to defend large territories and exclude sub-dominant individuals, resulting in sub-dominants collecting in lower quality habitats, defending smaller territories, and thus occurring at higher density^34^. Higher density in poorer quality landscapes combined with shapes of fecundity-density curves meant that in good quality landscapes, density fell on the upper, flatter parts of the curves whereas in poorer quality landscapes, density fell on the lower, steeper part of curve (Fig. 2). This led to an expected positive association of average fecundity with landscape quality (Fig. 3b) but also led to a negative association between the strength of density dependence and landscape quality; populations in higher quality landscapes tended to be more weakly regulated than those in poor quality landscapes (Fig. 3c).

Declines were more severe in landscapes where the strength of breeding density-dependence (*b*′) was small (Fig. 4). This negative relationship between trends and *b*′ matched the prediction from the hypothesis that wood thrush in the study region were primarily limited by availability of non-breeding habitat and declines can be primarily attributed to shifts in mortality-density curves caused by non-breeding habitat loss or other events in the non-breeding season. Other findings from this study and others provide additional support for the non-breeding limitation hypotheses^38^. In this study, I found that severe declines occurred in good quality landscapes and also that breeding density was low and density-dependence was weak in good quality landscapes. In another study, surveys conducted on a study site in NH from 1969 to 1998 showed the decline and extirpation of breeding wood thrush from apparently undisturbed habitat^39,40^. The authors this to forest maturation, which is a possible explanation, but wood thrush inhabit mature forest in other regions^41,42^. In general, we do not expect to observe low density and declines in good quality, undisturbed breeding habitat if breeding habitat is limiting^38^.

The finding of non-breeding limitation in the northeastern region is consistent with results from a network population model in which parameters for density–dependent processes in breeding and non-breeding were estimated by fitting a spatial network model to connectivity data and patterns of declines across the entire breeding range. The network model study concluded that, despite habitat loss and fragmentation in both seasons, declines were primarily caused by non-breeding habitat loss and fragmentation and the wood thrush population was largely limited by the availability and/or condition of non-breeding habitat^12^. Non-breeding limitation does not mean that breeding season events have had no effect on the population dynamics of wood thrush and the network model showed that, while loss of breeding habitat caused small declines overall, they could be quite large in the region affected^12^. Even in a primarily non-breeding limited system, fecundity-density curves are expected to shift in response to increased fragmentation of breeding habitat or changes in the abundance of nest parasites or predators. Observed correlations between local breeding declines and local breeding habitat loss^9^, between local declines and increases of nest parasites in the landscape^43^, and findings that fecundity contributes strongly to variation in local breeding population growth rates^10,11^ are entirely consistent with the Sutherland two-season framework but cannot be interpreted as evidence of breeding limitation or indication of causes of global declines. The implication for conservation of wood thrush is to focus management actions on preserving or restoring non-breeding habitat in Central America but also to prevent further fragmentation in breeding habitat to prevent intensification of density dependence in the breeding season^12^.

I found an unanticipated, positive relationship between the magnitude of trends and landscape quality, i.e., declines were more severe in high quality landscapes (Fig. 1b). I term this pattern an inverse buffer effect, since it is the opposite of a buffer effect where population trends are negatively related to quality and the largest declines or increases are found in the poorest quality regions^31,32^. Although the inverse buffer effect is a surprising and novel pattern that has not been previously reported, it is not clear whether the pattern has biological significance or is simply a peculiarity of this system. In wood thrush, the inverse buffer effect resulted from the confluence of several factors. First densities were low in breeding landscapes, which presumably was a result of the population not being limited by breeding habitat. Second, low densities combined with the convex shape of density dependent fecundity led to a negative relationship between *b*′ and quality. Third, because declines were primarily caused by events in the non-breeding season, there was also a negative relationship between trends and *b*′. These two negative relationships combined to give the positive relationship between magnitude of trends and landscape quality; the inverse buffer effect. Finding a large-scale inverse buffer effect in the breeding range does not indicate, by itself, that non-breeding habitat is limiting and is the cause of declines. In fact, we might also expect an inverse buffer effect if the system were breeding limited and declines caused by loss of breeding habitat because in this case, trends would be positively associated with *b*′ and breeding densities would be high which combined with the convex shape of density dependent fecundity would tend to produce a positive relationship of *b*′ and quality. Two positive relationships could also lead to an inverse buffer effect. In general an inverse buffer effect can arise if population trends are driven by habitat loss in the season that limits the population and density dependence is convex.

The opposite pattern, large scale buffer effects, have been observed in both seasons in an increasing Icelandic-breeding population of black-tailed godwits, *Limosa limosa islandica* ^32,44^. To observe this pattern within the framework presented here and assuming that density-dependence is convex requires that the population changes be driven primarily by events in the season that does not limit the population. While this perhaps seems unlikely, it may be possible for an increasing population, such as the godwits. However, to really use this framework to understand a buffer or inverse buffer effect in godwits or other species would require knowledge of the shapes of density dependence in that species.

This study presents an incomplete picture of wood thrush dynamics. Since forest patch size distributions were only available for states within the northeastern USA, the shapes of fecundity-density curves could only be estimated for eleven landscapes that cover only part of the breeding range. Also, since the fragmentation data as well as the field data used in the site-dependence model to estimate fecundity-density curves were both from the early 1990’s, this study was restricted to this time period and used BBS-derived trends and density estimates from the same time period. It is not known whether the inverse buffer effect and other findings exist outside of the spatial extent of this study or during other time periods. In addition, this study estimated the shape of nest survival-density curves and assumed that, although nest survival is only one component of fecundity, that the shape of the nest survival–density curve reflects the shape of the fecundity-density curve. This assumption can be justified because nest survival is the component of fecundity most likely to be strongly affected by forest patch size and studies have shown that more complete metrics of fecundity, i.e., number of offspring fledged per nest, were also higher in unfragmented landscapes or larger patches^4–6^. It is possible, however, that some other component of fecundity, such as post-fledging survival, might have a negative relationship with patch size and therefore alter the shape of the fecundity-density curve.

Examination of the changes of wood thrush populations over a longer time frame shows that in four of the six good-quality landscapes, there seems to be a steepening in decline rate or a reversal from increasing to declining population size in the late 1970s (Fig. S4). Although many other explanations can be advanced, it is possible that steepening occurred when density became low enough that the tipping points in the density-dependent fecundity were reached. It is however very difficult to understand the drivers of past declines due to the complexity. In any region, fecundity curves may have changed shape in response to increased nest-parasitism or fragmentation (or both) such that the tipping point moved above the density rather than the density dropping below the tipping point.

In summary, fragmentation of breeding habitat has been shown to reduce fecundity in many animal populations and thus is an important component of habitat quality for such populations. However, how fragmentation affects demographic processes, especially how it interacts with density-dependence, is not well understood. Here, I showed for wood thrush, a species whose fecundity is known to be sensitive to forest fragmentation, that fragmentation affects the shape of fecundity-density curves at a landscape scale. In high quality, less fragmented landscapes, fecundity-density curves were abruptly convex whereas in lower quality, more fragmented landscapes, fecundity-density curves were only slightly convex, almost linear in the poorest quality landscapes. Fecundity-density curves combined with landscape density and trend estimates from the BBS showed a negative association between the magnitudes of population trends and the realized strength of density dependence providing support for the hypothesis that wood thrush populations were not strongly limited by availability or condition of breeding habitat and that, at the species-level, the steep declines in have been primarily driven by loss of habitat or other events in the non-breeding season. Density in the breeding landscapes was generally low, especially in high quality landscapes and this led to a negative association between the strength of density dependence and landscape quality. The combination of the two negative associations between the magnitude of population trends and the realized strength of density dependence and between the strength of density dependence and quality provides an explanation for a surprising positive relationship between magnitude of trends and quality - an inverse buffer effect - population declines were most severe in the good quality landscapes. Determining the mechanisms of density dependence and the shape of density dependent demographic processes is vital to understand and make accurate predictions of large-scale population patterns and dynamics.

## Methods

### Landscapes and Landscape Quality

Forest patch size distributions for 13 states in the Northeastern USA were available from a special study on Landscape Dynamics conducted within the US Forest Service Northeastern Forest Inventory (https://www.fs.fed.us/ne/fia/studies/index.html). Patch size distributions were available as number of patches and total area within each of 8 forest patch size classes in all counties within the state (Fig. S2) and also as number and total area of core forest patches, where core forest was defined as forest > 30 m from an edge (Fig. S2). I defined eleven *landscapes* using state boundaries. Nine of these landscapes were one state each labeled by their standard postal abbreviations but the two smallest states (RI and DE) did not contain enough routes from the BBS data (see below) to estimate population trends and so were merged into neighboring states. Landscape CTRI was the combination of Connecticut (CT) with Rhode Island (RI) and MDDE was the combination of Maryland (MD) with Delaware (DE; Fig. 1a). *Landscape quality* was defined as the total area of core forest divided by the total area of all forest and thus was a measure of the degree of fragmentation within the landscape. Since the forest inventory data was generated from the 1992 National Land Cover Dataset 1992 (NLCD1992; uses satellite data circa 1990), the landscape quality metric represents the fragmentation of the landscape in the early 1990s. Patch size distributions for all counties within each landscape were used to estimate a patch size distribution for the whole landscape by assuming patches within a size class for a given county were equal to each other in area (i.e., by dividing total area in a county/class by the number of patches in that county/class).

### Landscape Population Trends and Density of Wood Thrushes

*Population trends* in Wood Thrush were estimated from the North American Breeding Bird Survey (BBS)^8^ by applying a hierarchical Bayesian model^15,45^ to estimate population abundance indices for each landscape from 1966 to 2015 (Fig. S4). Population trends in 1992 $|{\rm{\Delta }}n|$ were estimated as the average annual proportional change in population index between 1987 and 1997. A model and parameter values from the Partners in Flight Population Estimates Database was used to convert average birds per BBS route to an estimate of population size, the number of individuals^46^. *Population density*, was calculated at the landscape scale as the population size divided by 2 divided by the total forest area (in hectares) within the landscape to represent an estimate of the number of breeding pairs per ha.

To test for buffer or inverse buffer effects, I conducted a Pearson’s linear correlation test between magnitude of trend and landscape quality. A negative correlation (bigger trends in lower quality landscapes) indicates a buffer effect whereas a positive correlation (bigger trends in higher quality landscapes) indicates an inverse buffer effect.

### Site dependence model

A field study conducted by Hoover *et al*.^7^ in the early 1990s in Pennsylvania, US showed that nest survival increased with forest patch size^7^. Using the function *nls* in the R programming language^27^, I found the least-square estimates of the parameters (${f}_{MAX}$ and ${s}_{1/2})\,\,$that gave the best fit of a saturating curve of the form $f={f}_{MAX}s/({s}_{1/2}+s)$ to the nest survival data from the Hoover *et al*. study^7^. This resulted in the following model for nest survival, *f*, as a function of forest patch size, *s* in ha (Fig. S3a),

1 $$f(s)=\frac{0.85s}{42+s}$$

Hoover *et al*.^7^ also reported that nest density was higher in smaller patches than in larger patches; average nest density in patches <80 ha of 0.37 nests ha^−1^ and 0.11 nests ha^−1^ in larger patches (>100 ha) and contiguous forest^7^. A review of other Wood Thrush breeding studies revealed that this pattern, of higher densities in smaller patches or in more fragmented forests, is commonly found^5–7,28,29^. I used *nls* to find the least-square estimates of the parameters (*l*, $\alpha$, *t*) that gave the best fit of a reverse sigmoid shaped model of the form $d=1\,+\,(l-1)/(1+\,{e}^{\alpha (s-t)})$ to densities estimated from the data reported in Hoover *et al*.^7^. This resulted in the following model of saturation density *d* as a function of patch size, *s*, in ha (Fig. S3b),

2 $$d(s)=1+\frac{0.13-1}{1+{e}^{0.18(s-13.4)}}$$

I developed a site-dependent habitat selection model that assumes that forest patches are preferentially occupied in order of decreasing size until each patch reaches a saturation density, *d*, where *d* is a function of patch size, s (eqn. 2).

If all patches of size class *a* (where *a* is also the mean size of patches within the class) and larger are fully occupied (i.e., at saturation density), the average nest survival of the population is given by,

$$F(a)=\frac{{\sum }_{s=a}^{sU}\,f(s)d(s)A(s)}{{\sum }_{s=a}^{sU}d(s)A(s)}$$

and the density of birds in the landscape is

$$D(a)=\frac{{\sum }_{s=a}^{sU}d(s)A(s)}{{\sum }_{s=sL}^{sU}A(s)}$$

where *f*(*s*) is the average nest survival in a patch of size *s* (eqn. 1), *d*(*s*) is the saturation density of patch of size *s* (eqn. 2), *A*(*s*) is the total amount of area in size class *s*, and *sU*, *sL* are the maximum and minimum size classes in the landscape.

I generated values of *F*(*a*) and *D*(*a*) for all forest size classes from *sL* to *sU* and then used *nls* to fit the generalized Beverton-Holt (B-H) function^30^, $F=\frac{{F}_{max}}{1+{(D/K)}^{\gamma }}$ and estimate parameters *F*_*max*_, *K*, and for each landscape. *F*_*max*_ is the maximum nest survival, *K* is the density at which *F* is half of *F*_*max*_ and γ is the *abruptness* parameter that controls the shape of the curve. I calculated the *tipping point* of each curve as the point where the slope was changing most rapidly i.e., where the absolute value of the second derivative of *F* with respect to *D* was maximized. High goodness of fit for the B-H functions was verified visually by plotting the site-dependence model generated data versus the fitted B-H function.

### Nest survival and strength of density dependence

The realized average nest survival for each landscape was the value of the B-H function at the estimated population density. The strength of density-dependence in each landscape, *b*′, was calculated as the magnitude of the slope of the B-H function at the estimated population density. The magnitude of declines, $|{\rm{\Delta }}n|,$ were plotted against *b*′ and examined visually for trends. Sutherland’s two-season models^13,14^ showed that if trends are primarily driven by habitat change or events in the breeding season then we expect a positive relationship between magnitude of declines, $|{\rm{\Delta }}n|$ and *b*′, specifically we expect $|{\rm{\Delta }}n|\propto {b}^{^{\prime} }/({b}^{^{\prime} }+{d}^{^{\prime} })$, but if trends are primarily driven by habitat change or events in the non-breeding season then we expect a negative relationship between $|{\rm{\Delta }}n|$ and *b*′, specifically we expect $|{\rm{\Delta }}n|\propto {d}^{^{\prime} }/({b}^{^{\prime} }+{d}^{^{\prime} })$. In this case, trends were negatively related to *b*′ and so the best fit of the latter model was found assuming a constant value for the unknown strength of non-breeding density-dependence, *d*′.

All calculations and statistical analysis were performed using the R Programming Language^27^.

### Data Availability

All data used in this study are publicly available through the websites referenced.

## Electronic supplementary material

Supplementary Figures
