# Supplementary material for: The shape of density dependence in fragmented landscapes explains an inverse buffer effect in a migratory songbird
Source: Sci Rep. 2017 Nov 6;7:14522. doi: 10.1038/s41598-017-15180-4 (PMC5674021; doi:10.1038/s41598-017-15180-4)

Supplementary Figures

The shape of density dependence in fragmented landscapes explains an inverse buffer effect in a migratory songbird.

Caz M. Taylor

Department of Ecology and Evolutionary Biology, Tulane University, New Orleans LA 70118

Figure S1. Fecundity (red line) declines with population size (or density) and strength of breeding density-dependence, *b′* is the slope. The rightmost blue line is the non-breeding season mortality curve, which increases with population size. The strength of non-breeding density-dependence, *d′,* is the slope. The expected equilibrium population size is the point where the red and blue lines intersect. Non-breeding habitat loss causes the mortality curve (blue) to shift towards the left, which causes a decline in the equilibrium population size. When breeding density-dependence is weak (a), the population decline is larger than when breeding density-dependence is strong (b). Adapted from Sutherland (1996; *Proc. R. Soc. B* **263,** 1325–1327).

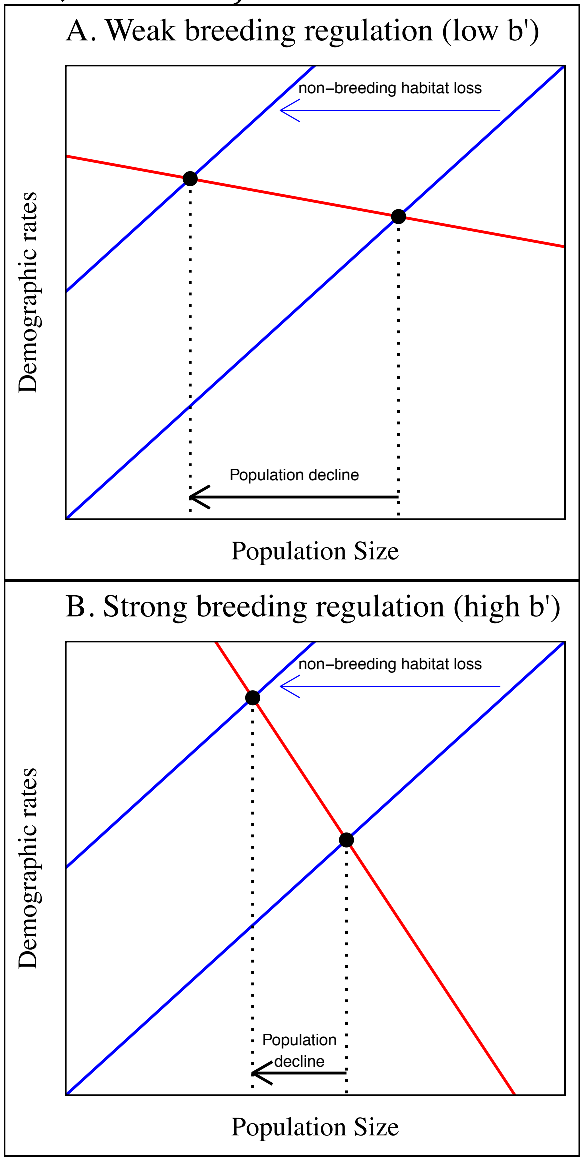

Figure S2. Forest patch size distributions for each of the eleven landscapes. Bar heights represent total forest area in class. Light green areas are area of core forest (forest that is > 30m from edge) in class. From a study on Landscape Dynamics conducted within the US Forest Service Northeastern Forest Inventory (https://www.fs.fed.us/ne/fia/studies/index.html). Landscapes are arranged (left to right, top to bottom) from low to high quality.

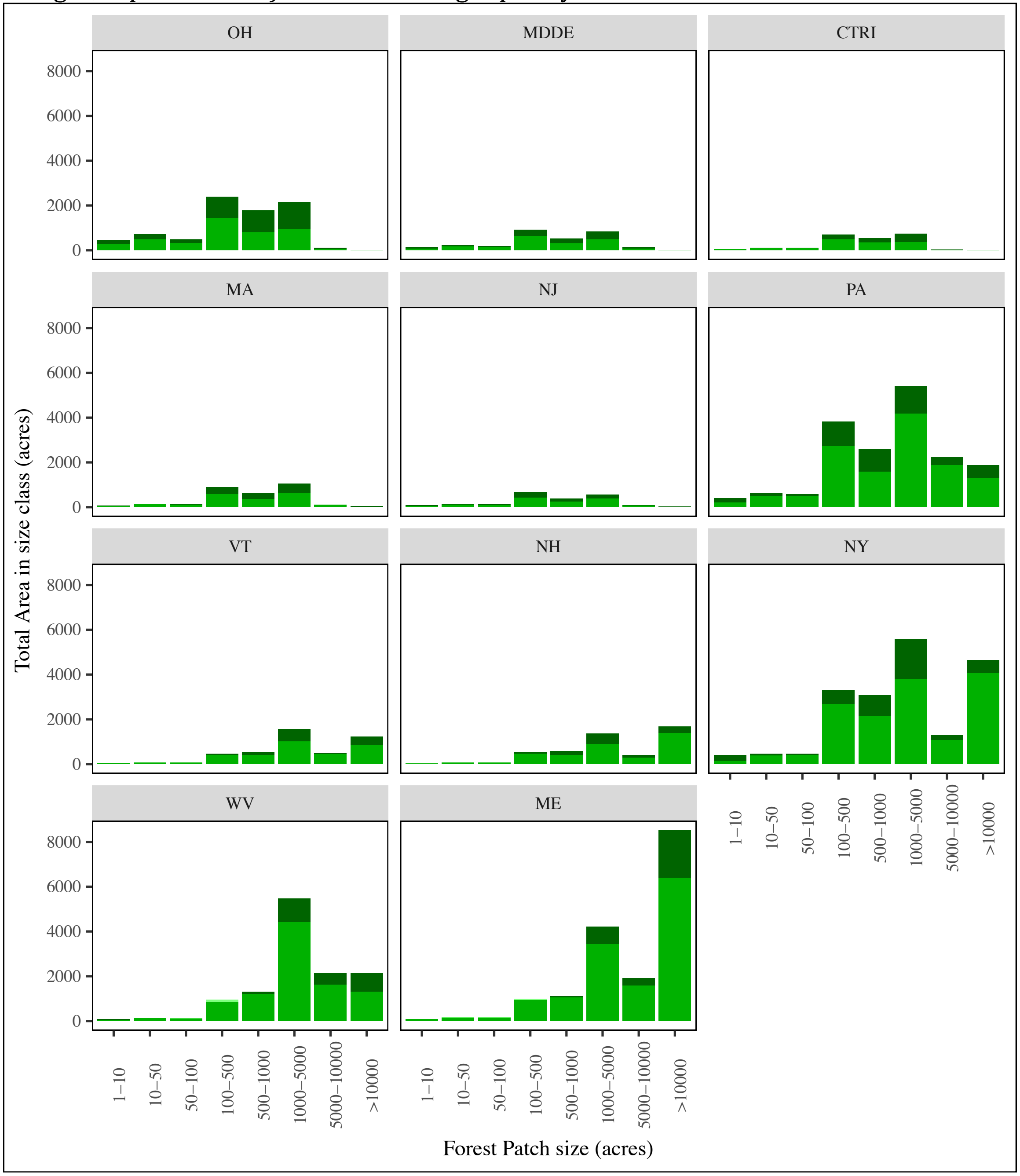

Figure S3. (a) Nest survival (probability) of wood thrush as a function of forest patch size. Data (points) from a field study in 1991 and 1992 in Pennsylvania, USA (Hoover, J. P., Brittingham, M. C. & Goodrich, L. J. 1995 *The Auk* **112,** 146–155). Solid line is the fitted model (eqn. 1, methods) of the expected nest survival in a patch of a given size. (b) Points are estimated densities (nests ha^{-1}) averaged from two years of field data in patches of different sizes estimated from the data reported in the same study. Solid line is fitted model (eqn. 2, methods) of the maximum or saturation density in a patch of a given size.

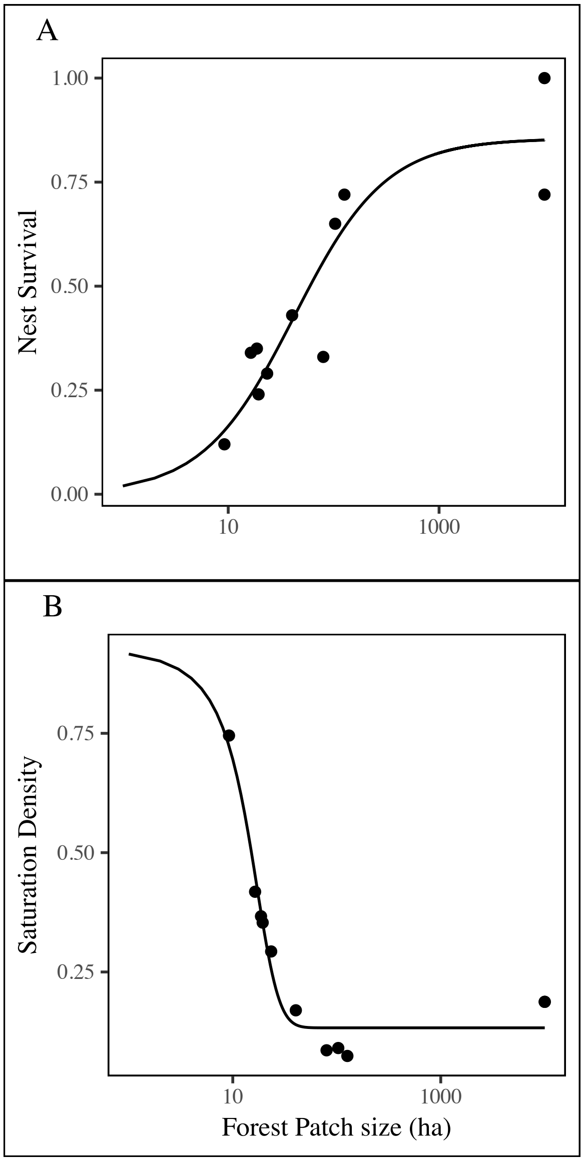

Figure S4. Population index of Wood Thrush in each of the eleven landscapes from 1966 to 2015 estimated using hierarchical Bayesian models from the North American Breeding Bird Survey (Link, W. A. & Sauer, J. R. 2002. *Ecology* **83,** 2832–2840) with fitted loess curve. Landscapes are arranged (left to right, top to bottom) from low to high quality.

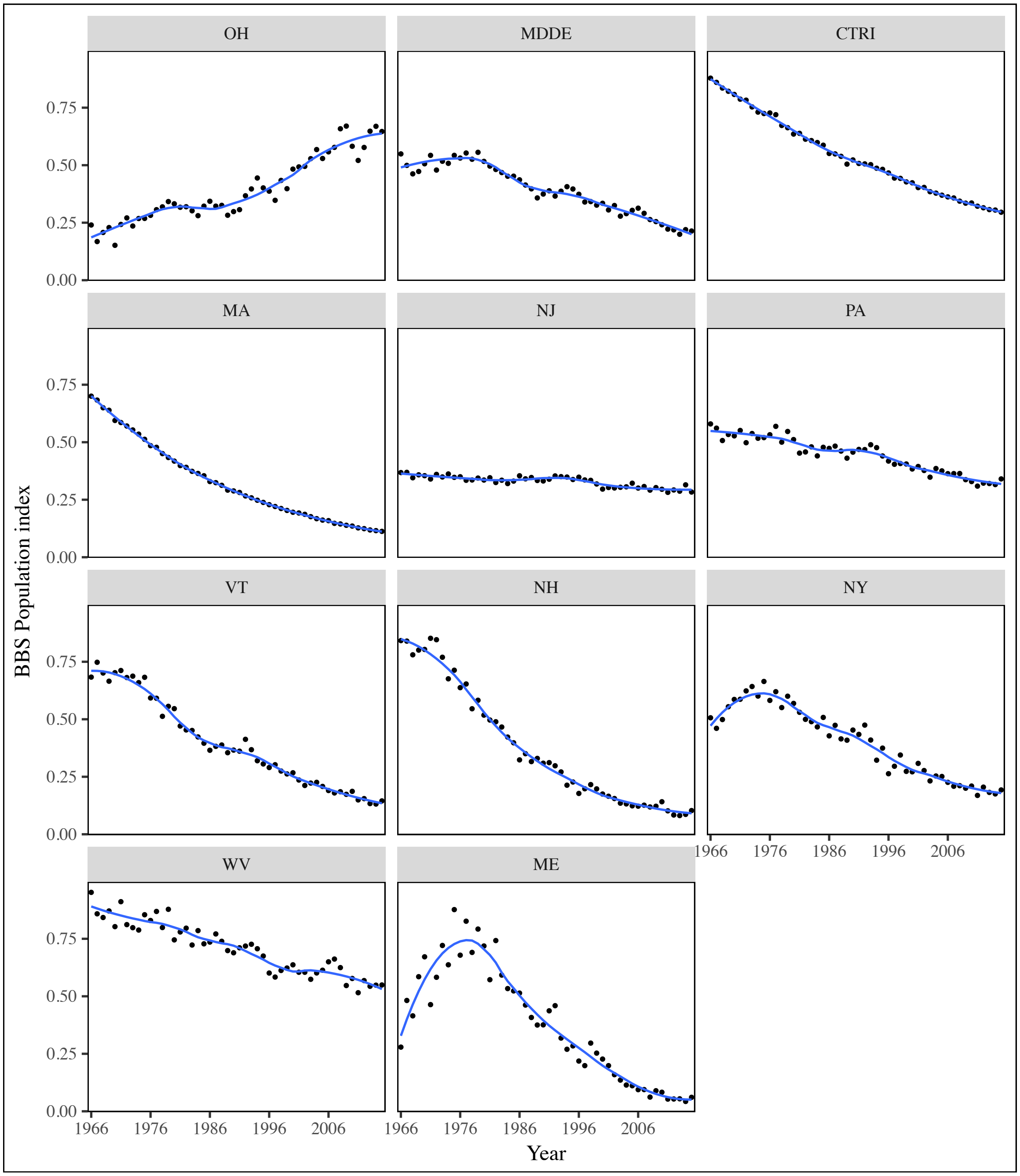

Supplement: Supplementary file 1 — Supplementary Figures [file 41598_2017_15180_MOESM1_ESM.pdf]
